# Finite element analysis of clear aligner with overhanging attachments and extended gingival coverage for interdental space closure 

**DOI:** 10.3389/fbioe.2025.1636262

**Published:** 2026-01-16

**Authors:** Guojin Zeng, Xiaoqing Ma, Dan Lin

**Affiliations:** 1 Shanghai University of Medicine and Health Sciences Affiliated Zhoupu Hospital, Shanghai, China; 2 Intelligent inspection and diagnostics health service platform, Shanghai University of Medicine and Health Sciences, Shanghai, China; 3 School of Stomatology, Xuzhou Medical University, Xuzhou, China; 4 Department of Orthodontics, Shanghai Xuhui District Stomatological Hospital, Shanghai, China

**Keywords:** clear aligners, finite element analysis, overhanging attachment, bodily movement, interdental space closure

## Abstract

**Objective:**

Overhanging (OH) attachments were modified clear aligner (CA) attachments with an extended portion toward the root to apply force closer to the center of resistance and enable greater control over root movement, resembling power arms. This study investigated the biomechanical effects of OH attachment and partially gingival extension of CA trimline on canine movement during the closure of extraction space via finite element analysis (FEA).

**Methods:**

CBCT data of an adult with Angle Class I molar relationship and mild anterior crowding was applied for comparing the biomechanical effects of three attachment types (no attachment, vertical, OH) and four trimline designs (partially buccal/lingual gingival coverage). Periodontal ligament (PDL) hydrostatic stress, tooth displacement, rotational center position, and CA stress distribution were assessed via FEA.

**Results:**

OH attachment induced increased tooth displacement and PDL hydrostatic stress (95.5 kPa) compared to regular vertical attachment (53.1 kPa) in achieving root-controlled canine movement. OH attachment combined with a buccolingual gingival extension of CA trimline on 2–6 facilitated the most translational canine movement and lowest ratio of mesio-apical to disto-occlusal displacement (0.466, compared to 0.506 in group with no attachment and trimline extension), while simultaneously avoiding excessive aligner deformation and stress concentration.

**Conclusion:**

Overhanging attachment combined with partial gingival extension of CA trimline significantly enhanced the orthodontic force for premolar extraction cases involving space closure between canines and molars, as a more efficient and feasible design for canine bodily movement.

## 1 Introduction

Clear aligners (CA) had been increasingly being utilized in orthodontics owing to their comfort, aesthetic appeal, and ease of cleaning (Weir, 2017). Despite their popularity, challenges still remained in managing intricate tooth movements, including torque (Cheng et al., 2022a), rotation (Seo et al., 2021), and bodily movement (Zhu et al., 2022). Specifically, during the closure of extraction spaces, torque controlling was critical to the translation of tooth and prevention of undesired tipping and rotation (Cheng et al., 2022a; Seo et al., 2021; Zhu et al., 2022).

The application of attachments was a common approach to facilitate more effective control of tooth movement (Je et al., 2023; Demir, 2024). In CA orthodontics, the standard design for closing extraction spaces involved placing attachments on canines (3) and posterior teeth (5/6/7) (Cheng et al., 2022a; Jiang et al., 2020; Liu et al., 2021; Liu L. et al., 2022; Liu J. Q. et al., 2022; Cheng et al., 2022b; Wang et al., 2022) and other auxiliaries, such as traction (Pu et al., 2022) and power ridges (Hong et al., 2024). These designs adjusted torque and balanced stress to precisely optimize tooth movement (Liu J. Q. et al., 2022; Wang et al., 2022; Xia et al., 2022). Root control attachments were also designed to modulate torque, including asymmetrical attachments with opposite orientations (Gomez et al., 2015; Yokoi et al., 2019) and overhanging attachments (Hong et al., 2021). An overhanging (OH) attachment was a modified CA attachment with an extended portion toward the root to apply force closer to the center of resistance and enable greater control over root movement, resembling a power arm (Hong et al., 2021). First reported in 2021 (Hong et al., 2021), an OH attachment was designed to induce bodily movement of the incisor and close scattered diastema of anterior teeth. A latest study in 2025 (Hong et al., 2025) reported the repair of gingival recession via better controlling root movement of lower incisor using an OH attachment. So far, there had been limited researches on OH attachments and their application in other tooth positions.

The apical extension of OH attachment necessitated the gingival extension of CA for full coverage. Extension of the trimming edges of CA had been reported to enhance control over tooth movement and improve therapeutic outcomes (Elshazly et al., 2023; Elshazly et al., 2022; Elshazly et al., 2024a; Elshazly et al., 2024b), as summarized in a recently published systematic review (Nakornnoi et al., 2024). However, despite of the widely acknowledged advantages of extended trimline, the remodeling of gingival soft tissues did not synchronize with tooth movement, and a full gingiva-covering trimline design compromised the aesthetics and comfort of CA. As a result, such trimline extensions have not gained widespread popularity in clinical practice. Reducing the gingival coverage from full to partial might avoid compromising the aesthetics and comfort of CAs, but whether equivalent effectiveness could be achieved remained unknown. Existing studies had focused on integral extension of CA trimline, with a lack of comparative research on the effects of region-specific extension of CA. Furthermore, the combined effects and biomechanical behavior of OH attachments with gingival extension of CA in closing extraction spaces remained to be explored.

In this study, finite element analysis (FEA) was employed to explore the biomechanical effects of different attachment designs (vertical/OH) combined with different gingival extension designs (buccal/lingual covering different regions) during closing extraction spaces. The effects on tooth movement, PDL stress distribution, and appliance deformation were systematically analyzed and compared to improve the effectiveness of CA and minimize adverse effects during closing extraction spaces. This study could provide guidance for future design of CA to improve the orthodontic effectiveness.

## 2 Materials and methods

### 2.1 Acquisition of dentition

The use of patient imaging data in this research was in accordance with institutional ethical standards, following informed consent and approval by the Ethical Committee of the Shanghai Xuhui District Dental Center, Shanghai, China. CBCT data of a healthy adult’s dentition (with ANB angle of 4.8°, average growth pattern, Angle Class I molar relationship, and mild anterior crowding) with a slice thickness of 0.625 mm (GE Healthcare, Buckinghamshire, England) was imported into Mimics software (Version 18.0, Materialise, Leuven, Belgium) for 3 days reconstruction.

### 2.2 Design of CAs and attachments

**FIGURE 1:**
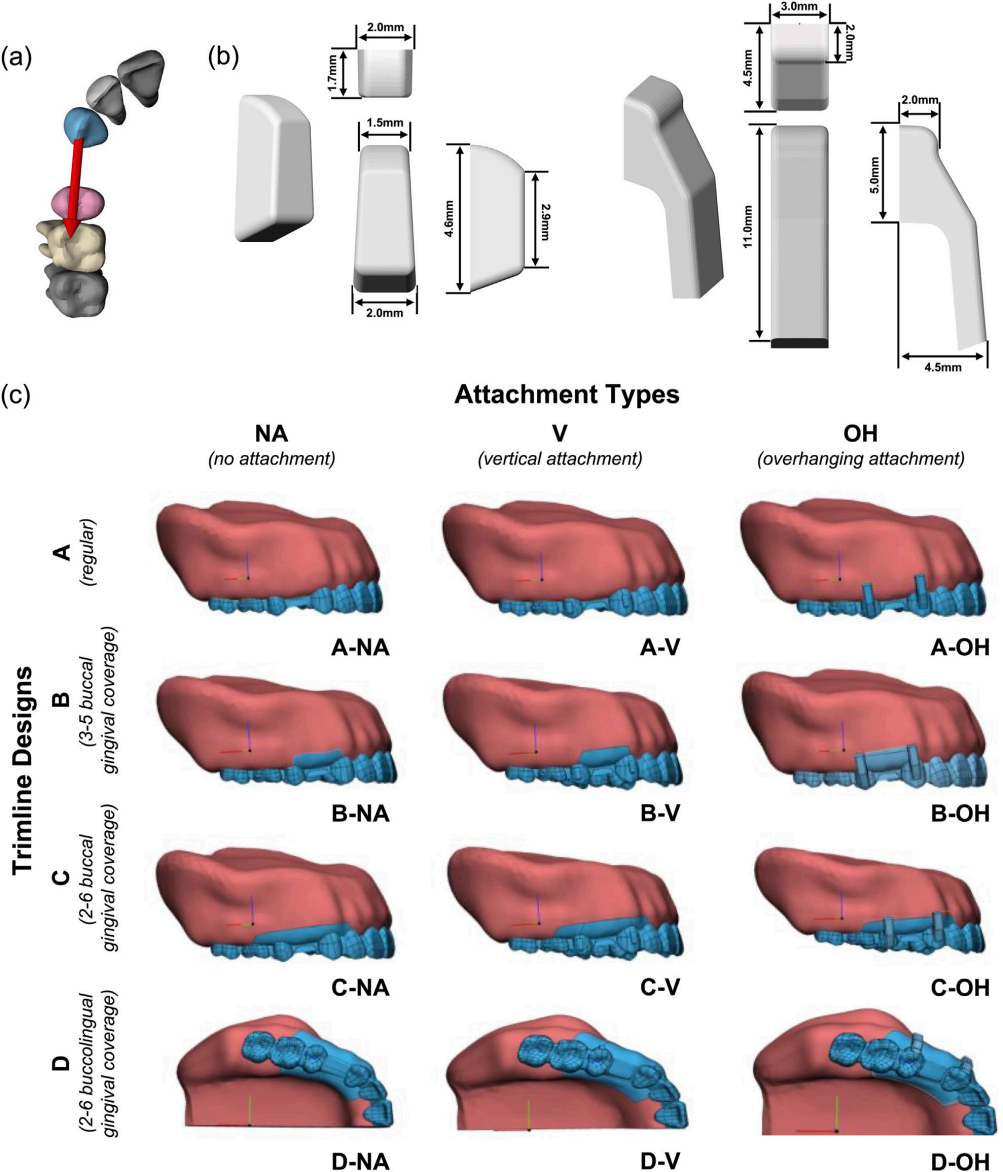
Design of CAs and attachments in Closing Extraction Spaces. **(a)** Designed tooth movement: distal movement of canines by 0.2 mm. **(b)** Shapes and dimensions of vertical attachment and OH attachment. **(c)** 12 groups of models for FEA comprising 3 attachment types and 4 CA trimline designs.

As illustrated in Figure 1a, CA with a thickness of 0.5 mm was designed on the reconstructed dentition model with the distal movement of canines (3) by 0.2 mm to provide orthodontic force. Attachments were adhered in the middle of the buccal surfaces of canines (3) and second premolars (5), and the attachment designs were categorized into 3 types: vertical wedge-shaped attachment (V), overhanging attachment (OH), and no attachment (NA). As shown in Figure 1b, vertical wedge-shaped attachments were designed with the wedge surface oriented towards the occlusal direction; OH attachments were designed with 6 mm extension towards gingiva.

CA trimline designs were divided into 4 types: (A) regular CA with unextended scalloped trimline; (B) CA with scalloped trimline and 5 mm buccal extension for gingival coverage at teeth 3∼5; (C) CA with scalloped trimline and 5 mm buccal extension for gingival coverage at teeth 2∼6; (D) CA with scalloped trimline and 5 mm buccolingual extension for gingival coverage at teeth 2∼6.

In total, 12 groups of models comprising 3 attachment types and 4 CA trimline designs were created and analyzed in this study, as shown in Table 1 and Figure 1c.

**TABLE 1:** 12 groups of models for FEA in this stud**y**, comprising 3 attachment types and 4 CA trimline designs.

Group	Trimline designs	Attachment types
A-NA	regular CA with unextended scalloped trimline	no attachment
A-V	regular CA with unextended scalloped trimline	vertical wedge-shaped
A-OH	regular CA with unextended scalloped trimline	overhanging
B-NA	CA with scalloped trimline and 5 mm buccal extension at teeth 3∼5	no attachment
B-V	CA with scalloped trimline and 5 mm buccal extension at teeth 3∼5	vertical wedge-shaped
B-OH	CA with scalloped trimline and 5 mm buccal extension at teeth 3∼5	overhanging
C-NA	CA with scalloped trimline and 5 mm buccal extension at teeth 2∼6	no attachment
C-V	CA with scalloped trimline and 5 mm buccal extension at teeth 2∼6	vertical wedge-shaped
C-OH	CA with scalloped trimline and 5 mm buccal extension at teeth 2∼6	overhanging
D-NA	CA with scalloped trimline and 5 mm buccolingual extension at teeth 2∼6	no attachment
D-V	CA with scalloped trimline and 5 mm buccolingual extension at teeth 2∼6	vertical wedge-shaped
D-OH	CA with scalloped trimline and 5 mm buccolingual extension at teeth 2∼6	overhanging

### 2.3 Preprocessing of 3D reconstructed models

The reconstructed maxilla and dentition were then imported into 3-Matic Medical software (Version 9.0, Materialise, Leuven, Belgium) for further processing. Both first premolars (4) were removed to simulate subtractive tooth extraction. Alveolar bone was divided into cortical bone and cancellous bone, with 2 mm thickness of cortical bone. The alveolar bone was expanded outward by 2 mm to simulate the gingiva, and the space between the teeth and alveolar bone was expanded by 0.2 mm to simulate the PDL, according to typical parameters in existing orthodontic FEA studies (Canales et al., 2013; Cao et al., 2023; Çifter and Saraç, 2011).

3D reconstructed models of maxilla, dentition, gingiva, PDL, CA, and attachment in STL format were imported into the Geomagic software (3D-SYSTEM, United States) for surface smoothing, eliminating any potential geometric imperfections and noise. Subsequently, the output file was exported as IGES format and imported into Hypermesh software (Altair, United States) for preprocessing, including geometry repair, component organization, convergence experiment and shell meshing, material parameter setting, contact definition, and the application of boundary conditions. A symmetric dentition model with respect to the sagittal plane was adopted for analysis, with results ultimately presented as unilateral dentition.

### 2.4 Material properties

The material parameters used in this study were obtained from material suppliers and literature (Seo et al., 2021; Canales et al., 2013; Sarrafpour et al., 2013), as detailed in Table 2. PDL was modeled using a visco-hyperelastic-damage constitutive model (Wu et al., 2022; Natali et al., 2008). To reduce computational complexity while maintaining accuracy, the teeth were modeled as rigid bodies, as prior studies have demonstrated that the difference in predicted displacement between rigid-body and fully elastic models was less than 3%, and that tooth deformation under orthodontic forces was negligible compared to overall movement (Tamaya et al., 2021; Hamanaka et al., 2017; Kim et al., 2025).

**TABLE 2:** Mechanical parameters (elastic modulus and Poisson’s ratio) of the materials.

Material name	CA	Attachment	Gingiva	Cancellous bone	Cortical bone
Elastic Modulus/GPa	1250	1250	2.8	1370	13700
Poisson’s Ratio	0.36	0.36	0.4	0.3	0.3

### 2.5 Loading and boundary conditions

The interaction between CA and teeth was achieved via an interference fit, applying normal hard contact and tangential frictional sliding conditions, and a friction coefficient of 0.2. Tie constraints were applied between PDL and teeth, the outer layer of PDL and alveolar bone, alveolar bone and gingiva, teeth and gingiva (Natali et al., 2008; Hahn et al., 2009; Ortún-Terrazas et al., 2020; Huang et al., 2023; Goktas et al., 2011). The symmetrical model with respect to the sagittal plane was subjected to symmetrical displacement constraints.

### 2.6 Construction of 3D finite element model

**FIGURE 2:**
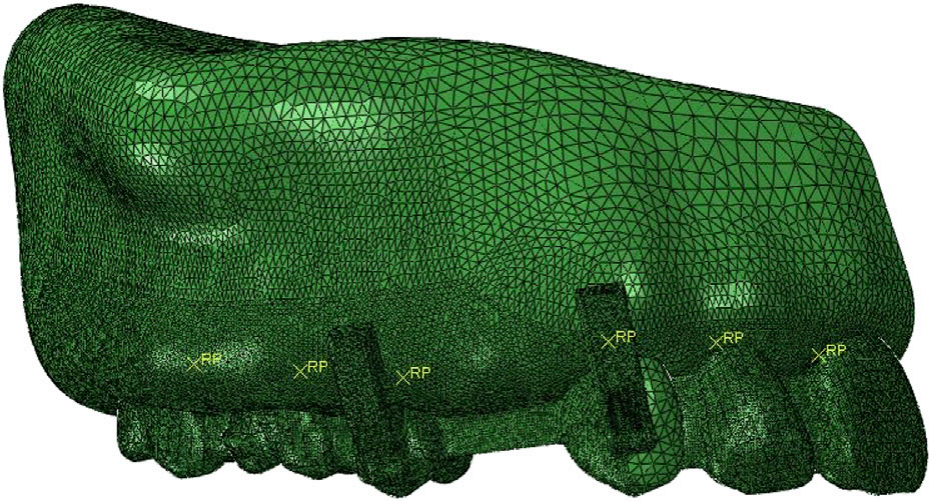
Meshed finite element model (A-OH group used as an example).

The general FEA software ABAQUS (DASSAULT, France) was employed as the solver and processor to perform a detailed biomechanical analysis. Element sizes of the models were determined via the convergence experiment to ensure a sufficiently refined mesh for the simulation, and the applied element types, sizes and counts, and node counts were listed in Table 3. As shown in Table 3 and Figure 2, CAs were simulated using S4R shell elements and an element size of 0.2 mm. Attachments were simulated using C3D10M elements with a size of 0.2 mm. Gingiva and PDL were modeled using C3D10H elements with a size of 0.2 mm. For alveolar bone, C3D10 elements were applied, with element sizes of 0.2 mm in regions sharing nodes with gingiva and PDL (Seo et al., 2021; AlKahlan et al., 2025), and gradual transition of element sizes to 2 mm at other regions. Dentition was simulated using R3D3 elements with a size of 0.2 mm in regions sharing nodes with PDL, and gradual transition of element sizes to 0.8 mm at other regions.

**TABLE 3:** Element types, sizes, counts, and node counts for the models. Element sizes were determined via the convergence experiment to ensure a sufficiently refined mesh for the simulation.

Component	Element type	Element size (mm)	Element count	Node count
Gingiva	C3D10H	0.2	184849	299435
PDL	C3D10H	0.2	94166	189790
Tooth	R3D3	0.2∼0.8	37957	19019
Cancellous Bone	C3D10	0.2∼2	169990	266400
Cortical Bone	C3D10	0.2∼2	109100	185611
Attachment	C3D10M	0.2	5783	10225
CA	S4R	0.2	37777	37535

## 3 Results and discussions

### 3.1 Hydrostatic pressure in PDL

The hydrostatic pressure exerted on the PDL served as an indicator of its tensile and compressive states. Exposure to tensile or compressive stress activated the internal vascular and neural elements of the PDL under their stress conditions, thereby triggering a series of biochemical reactions and activating osteoclasts and osteoblasts, which led to bone remodeling at the interface between the PDL and the alveolar bone. Typically, bone resorption occurred on the compression side, while bone formation took place on the tension side (Sarrafpour et al., 2013; Li et al., 2021).

**FIGURE 3:**
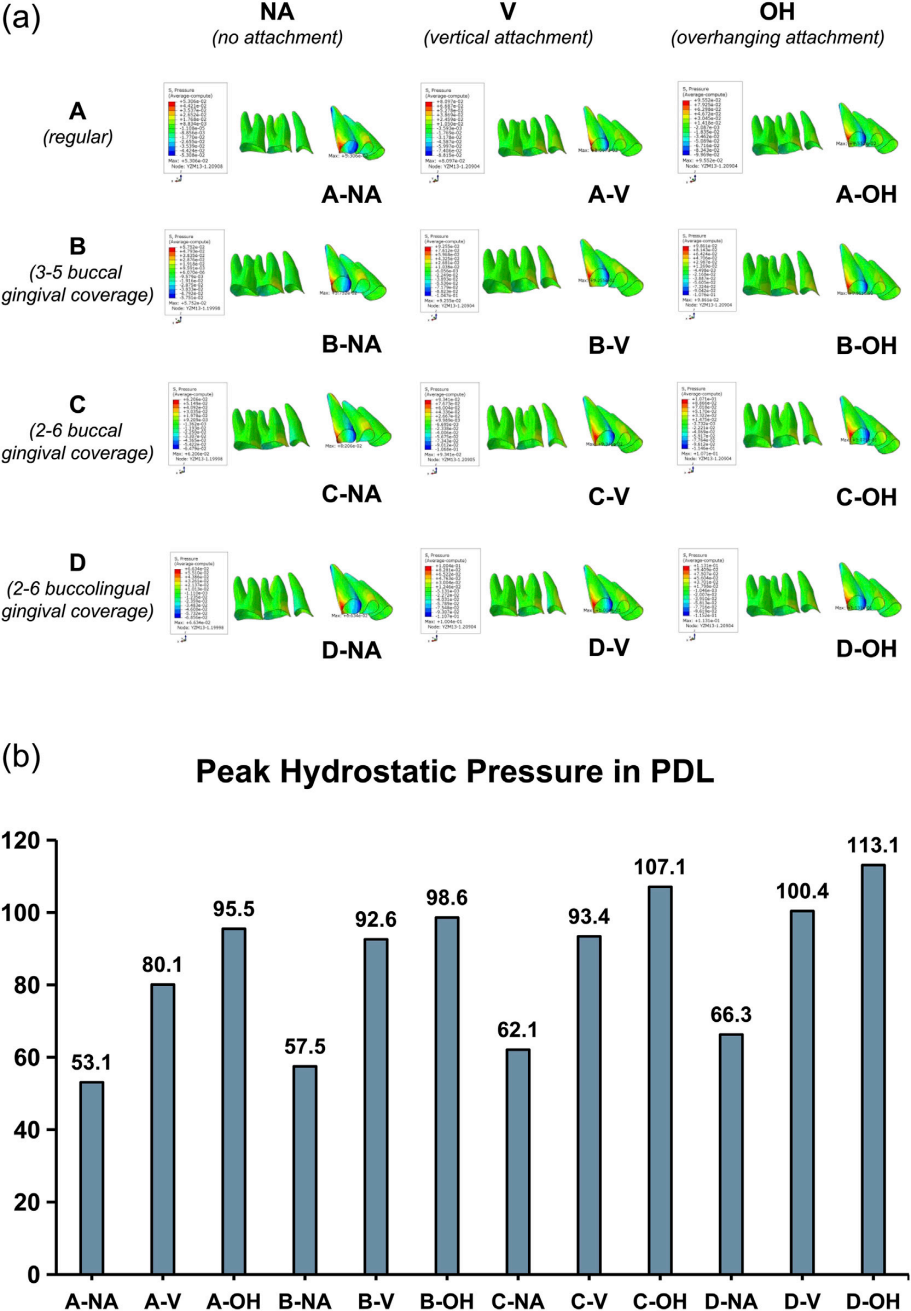
Hydrostatic pressure in PDL. **(a)** Heatmap of hydrostatic pressure in PDL, indicating compression (+) at disto-cervical and mesio-apical areas, and tension (−) at disto-apical and mesio-cervical areas. **(b)** Peak hydrostatic pressure in PDL of canine (unit: kPa). CA with 2–6 buccolingual gingival extensions and OH attachments exhibited the highest hydrostatic pressure in PDL.

As illustrated in the Figure 3a, it was evident that during the root control movement of canine (3) following the extraction of first premolar (4), the disto-cervical and mesio-apical areas of PDL near were subjected to compression (+), whereas the disto-apical and mesio-cervical areas were tension (−). The maximum and mean stress values for these regions were presented in Table 4. The comparison of the maximum hydrostatic pressure in the PDL was shown in Figure 3b. Under the same CA trimline design condition, the peak hydrostatic pressure in the PDL progressively increased from the no-attachment (NA) group, to the regular vertical attachment (V) group, and further to the overhanging attachment (OH) group, with the OH group exhibiting the highest pressure. Similarly, under the same attachment design condition, the peak hydrostatic pressure also increased sequentially from the conventional CA trimline (A), to the 3∼5 buccal extension (B), to the 2∼6 buccal extension (C), and to the 2∼6 buccolingual extension (D), with the D group presenting the maximum pressure. The application of regular attachments or OH attachments, as well as the buccal or lingual extended coverage of the CA, all enhanced the orthodontic force, thereby increasing the stress values in PDL. In all, the CA with 2∼6 buccolingual gingival extensions and OH attachments exhibited the highest hydrostatic pressure in PDL.

**TABLE 4:** Maximum and mean compressive (+) and tensile (−) stress in PDL of canine (unit: kPa).

Group	Location	Maximum		Mean	
		Mesial	Distal	Mesial	Distal
A-NA	Cervical	−52.0	53.1	−39.5	41.9
	Apical	53.0	−53.1	63.3	−41.1
A-V	Cervical	−88.2	81.0	−71.1	66.6
	Apical	73.3	−79.8	63.3	−70.1
A-OH	Cervical	−99.7	95.5	−83.3	78.0
	Apical	81.0	−88.0	65.5	−74.5
B-NA	Cervical	−57.5	57.5	−46.8	43.8
	Apical	56.2	−57.2	46.6	−47.6
B-V	Cervical	−104.7	92.6	−84.1	79.8
	Apical	79.8	−89.7	65.7	−73.6
B-OH	Cervical	−107.6	98.6	−87.3	84.5
	Apical	78.5	−90.6	67.3	−72.3
C-NA	Cervical	−62.1	64.8	−54.4	51.6
	Apical	60.8	−63.8	50.5	−51.7
C-V	Cervical	−106.8	93.4	−84.9	80.2
	Apical	79.2	−91.6	63.9	−73.9
C-OH	Cervical	−114.6	107.1	−96.2	89.5
	Apical	83.1	−96.0	71.0	−78.5
D-NA	Cervical	−68.6	66.3	−54.4	53.0
	Apical	61.0	−64.9	50.7	−51.2
D-V	Cervical	−110.7	100.4	−90.6	92.5
	Apical	83.6	−95.5	71.1	−79.2
D-OH	Cervical	−115.2	113.1	−96.9	95.3
	Apical	86.0	−91.2	72.8	−76.4

**FIGURE 4:**
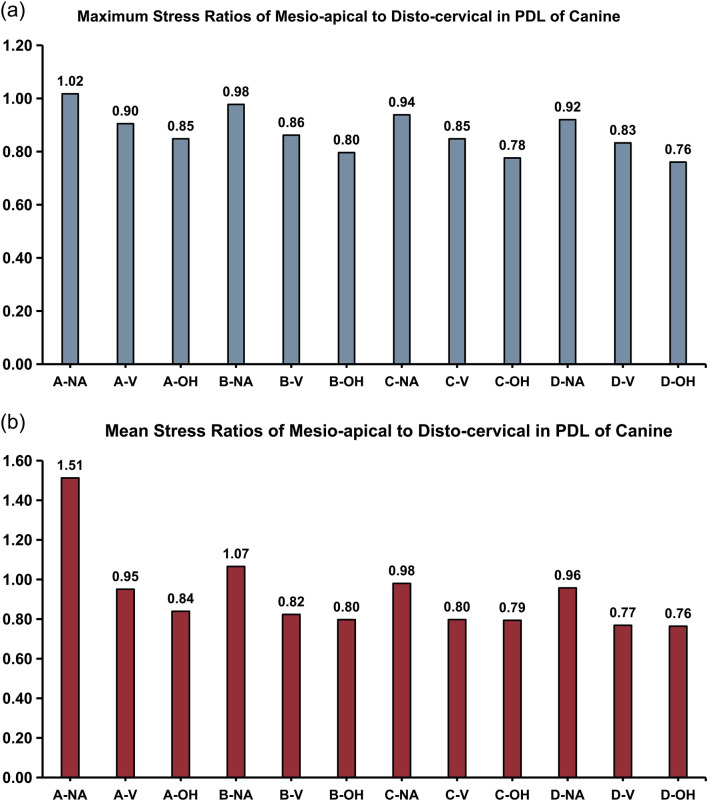
The ratios of mesio-apical to disto-cervical stress in PDL of canine, calculated using **(a)** maximum or **(b)** mean value. The closer the ratio was to zero, the closer the movement was to distal translation.

The ratio of mesio-apical to disto-cervical stress of PDL reflected the tipping extent of the tooth (Bi and Shi, 2023). In an ideal situation when the canine moved distally and translationally, both mesio-cervical and mesio-apical hydrostatic stress were tensile (with negative value or zero), and both disto-cervical and disto -apical hydrostatic stress were compressive (with positive value or zero), resulting in a zero or negative value of the ratio of mesio-apical to disto-cervical stress. Therefore, the closer the ratio of mesio-apical to disto-cervical stress was to zero or negative value, the closer the tooth movement was to distal translation. In this study, the ratios of mesio-apical to disto-cervical stress in PDL of canine in different groups were shown in Figure 4, calculated with maximum stress value (Figure 4a) or mean value (Figure 4b). With the same CA trimline design, the ratios progressively decreased from no-attachment (NA) group, to regular vertical attachment (V) group, and further to OH attachment group, with the OH group exhibiting the lowest ratio, indicating OH attachment was the most effective for achieving bodily tooth movement among the three attachment designs. Similarly, with the same attachment design, the ratios decreased sequentially from the conventional CA trimline (A), to the 3∼5 buccal extension (B), to the 2∼6 buccal extension (C), and to the 2∼6 buccolingual extension (D), with the D group presenting the minimum ratio. It could be concluded that the CA with 2∼6 buccolingual gingival extensions and OH attachments represented the optimal configuration for facilitating translational canine movement.

### 3.2 Tooth displacement

**FIGURE 5:**
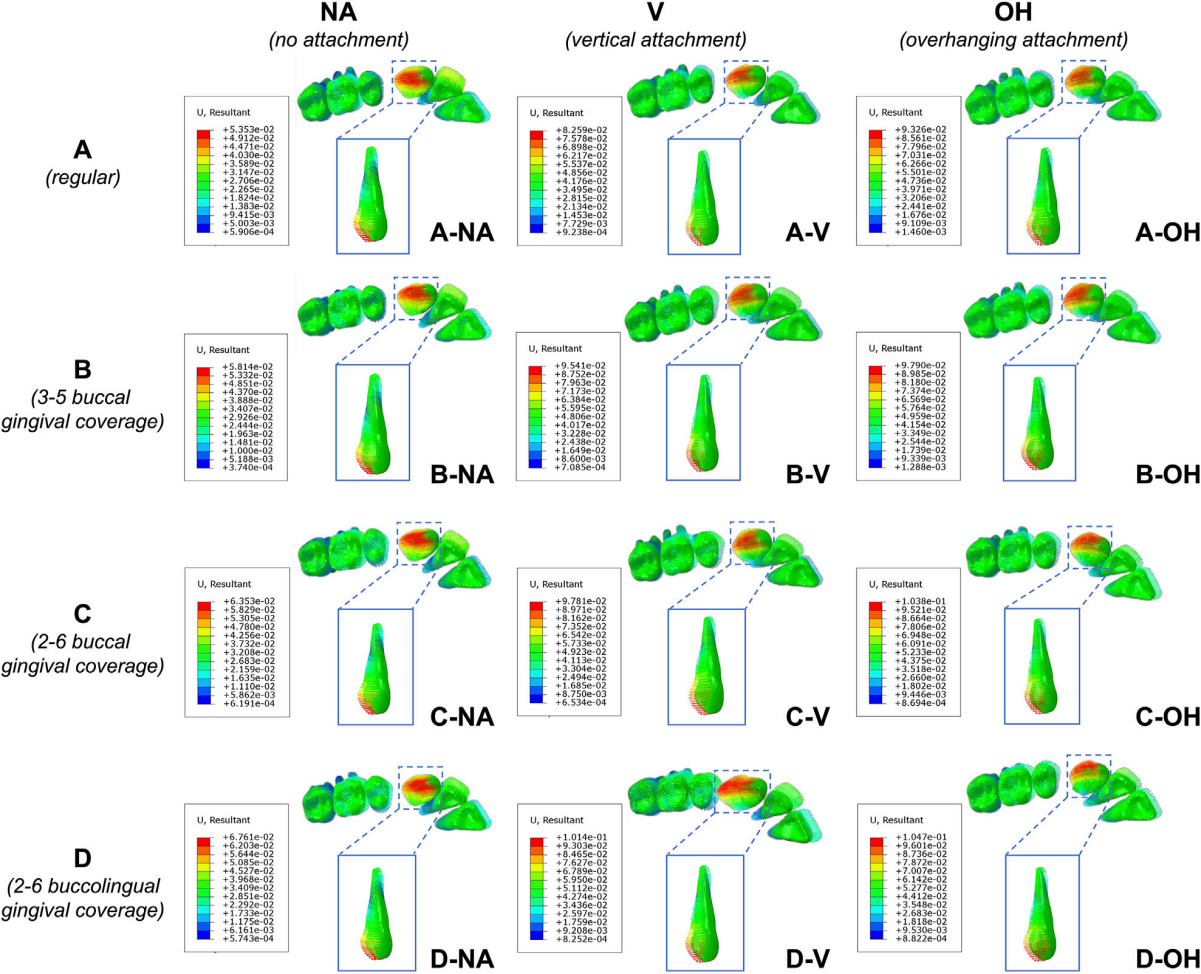
Heatmap of tooth displacement in vector diagram. Maximum values on heatmap scale revealed a trend consistent with hydrostatic pressure in PDL: OH > V > NA and D > C > B > A. **(A)** Regular CA; **(B)** CA with buccal gingival coverage from 3 to 5; **(C)** CA with buccal gingival coverage from 2 to 6; **(D)** CA with buccolingual gingival coverage from 2 to 6.

Figure 5 presented the heatmap of tooth movement in vector diagram. In all groups, distal inclination of canine was observed, accompanied with labial tipping of incisors and mesial inclination of molars, as an outcome of normal anchorage effect. As evidenced by the upper limits of the heatmap scale, the trend in tooth displacement aligned with the prior findings for PDL hydrostatic pressure, following the pattern OH > V > NA and D > C > B > A.

**FIGURE 6:**
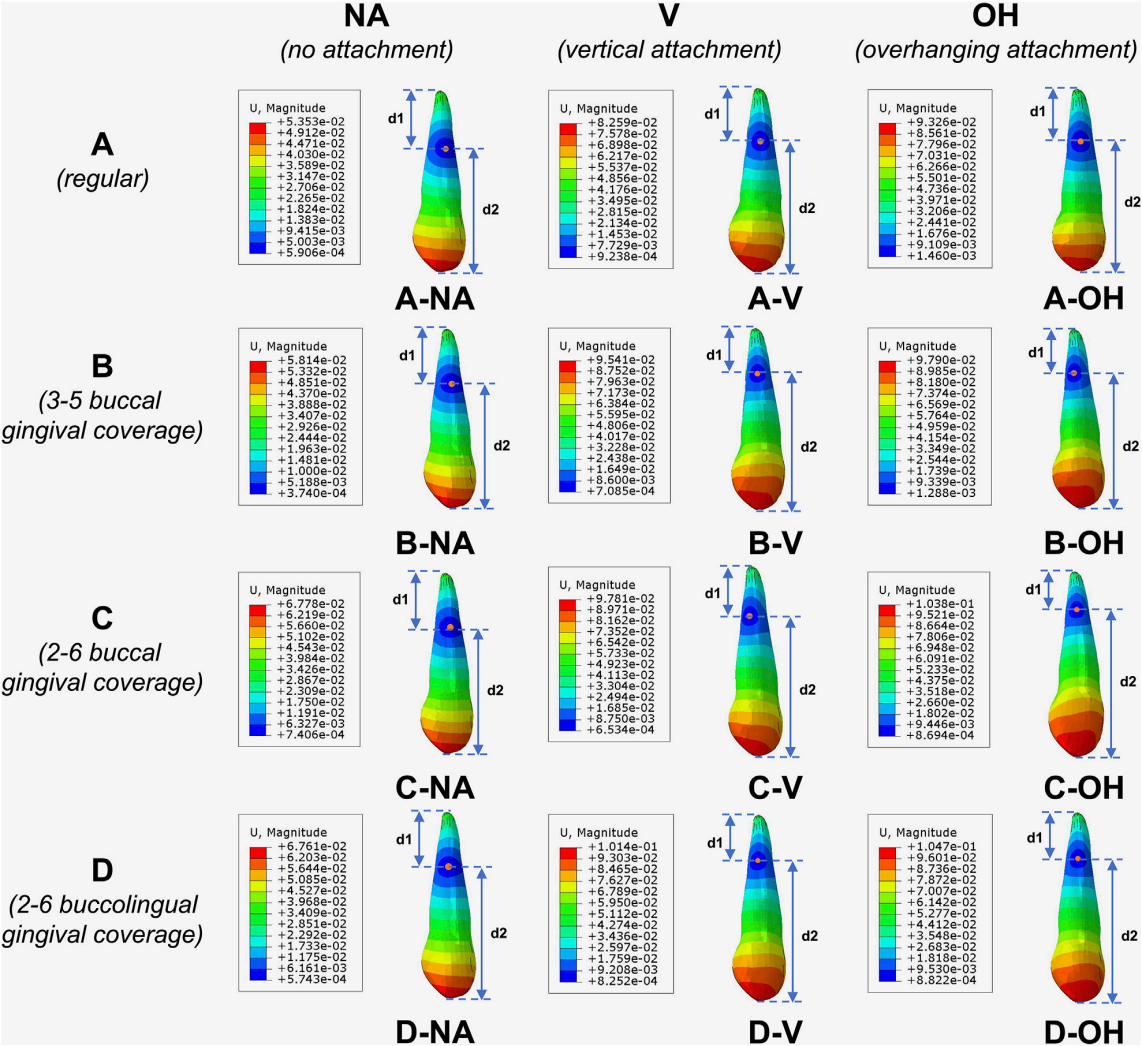
Heatmap of tooth displacement with contour. Point of minimum displacement (center of blue region) represented the center of rotation. **(A)** Regular CA; **(B)** CA with buccal gingival coverage from 3 to 5; **(C)** CA with buccal gingival coverage from 2 to 6; **(D)** CA with buccolingual gingival coverage from 2 to 6.

**FIGURE 7:**
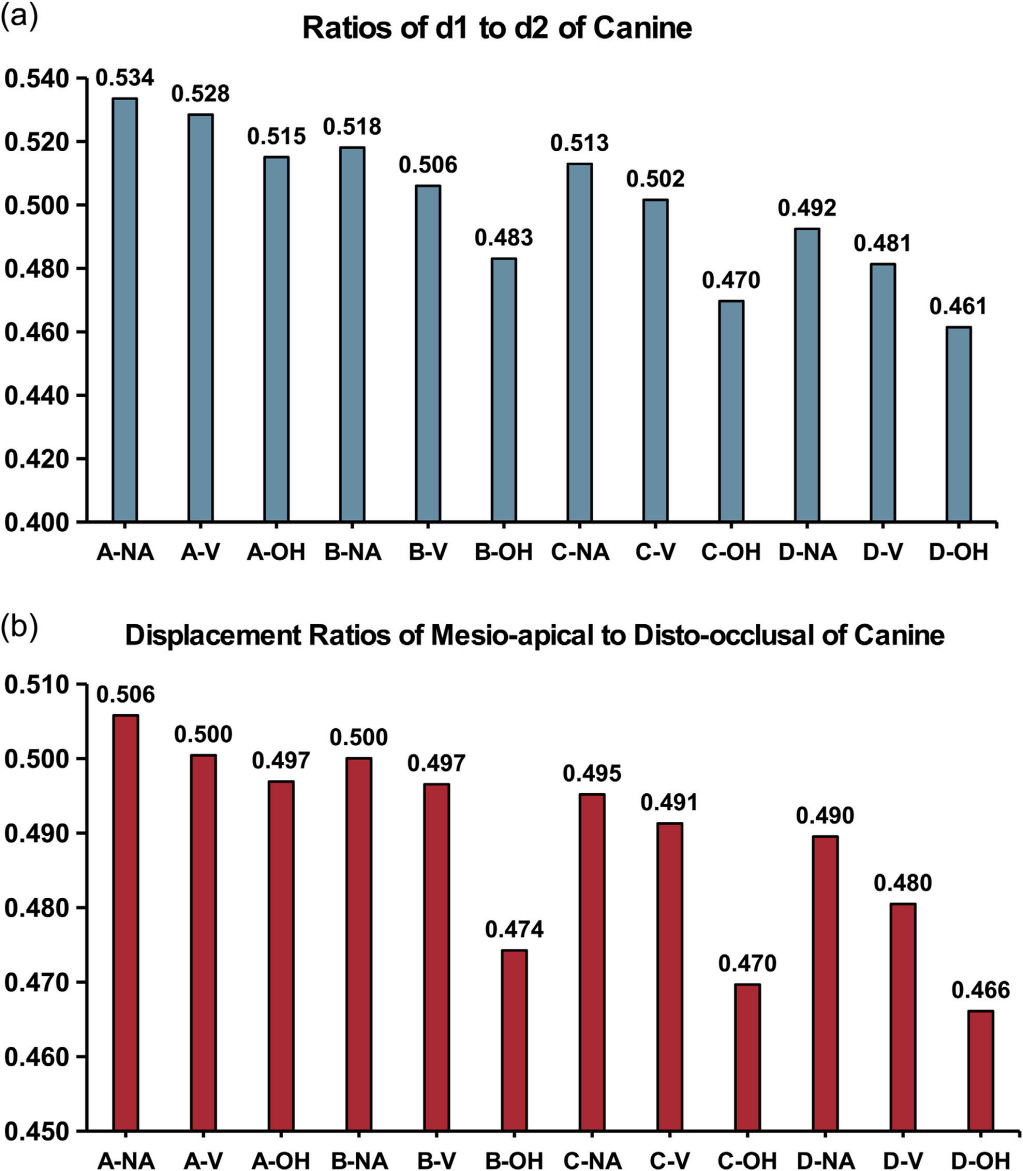
Degree of root control during canine movement. Ratios of **(a)** *d1/d2* and **(b)** mesio-apical to disto-occlusal displacement of canine. The smaller the ratios, the closer the tooth movement was to distal translation.

Figure 6 presented the heatmap of tooth movement with contour, where the point of minimum displacement (the center of blue region) represented the center of rotation of canine (Seo et al., 2021). Distances from root apex and tooth cusp to the rotational center were respectively designated as *d1* and *d2*. The smaller the ratio of *d1/d2*, the closer the rotational center was to the root apex. And a ratio of zero indicated translational movement of the tooth. The ratios of *d1/d2* in different groups were shown in Figure 7a. The ratio of mesio-apical to disto-occlusal displacement of canine (Figure 7b; Table 5) also reflected the degree of root control. The smaller the ratio, the closer the tooth movement was to distal translation (Liu L. et al., 2022).

**TABLE 5:** Mesio-apical and disto-occlusal displacement of canine (unit: mm).

Group	Mesio-apical displacement	Disto-occlusal displacement
A-NA	0.0532	0.0269
A-V	0.0709	0.0355
A-OH	0.0742	0.0369
B-NA	0.0561	0.0281
B-V	0.0784	0.0389
B-OH	0.0806	0.0382
C-NA	0.0620	0.0307
C-V	0.0796	0.0391
C-OH	0.0806	0.0378
D-NA	0.0639	0.0313
D-V	0.0835	0.0401
D-OH	0.0807	0.0376

With the same CA trimline design, the ratios of *d1/d2* progressively decreased from no-attachment (NA) group, to regular vertical attachment (V) group, and further to OH attachment group, with the OH group reaching the minimum value. Similarly, with the same attachment design, the ratios of *d1/d2* decreased sequentially from the conventional CA trimline (A), to the 3∼5 buccal extension (B), to the 2∼6 buccal extension (C), and to the 2∼6 buccolingual extension (D), with the D group presenting the minimum ratio. In all, D-OH group exhibited the smallest ratio of *d1/d2*. A similar trend was observed regarding the ratio of mesio-apical to disto-occlusal displacement of canine (Figure 7b; Table 5).

The CA with extended buccal and lingual gingival coverage and OH attachments effectively reduced the ratio of *d1/d2* to 0.461, which was beneficial for controlling tooth rotation and reducing the inclination of tooth during translational movement. Findings in this study were consistent with previous reports (Elshazly et al., 2023; Elshazly et al., 2022) suggesting that extending the gingival coverage of CA benefited root control movement.

### 3.3 Deformation of CA

**FIGURE 8:**
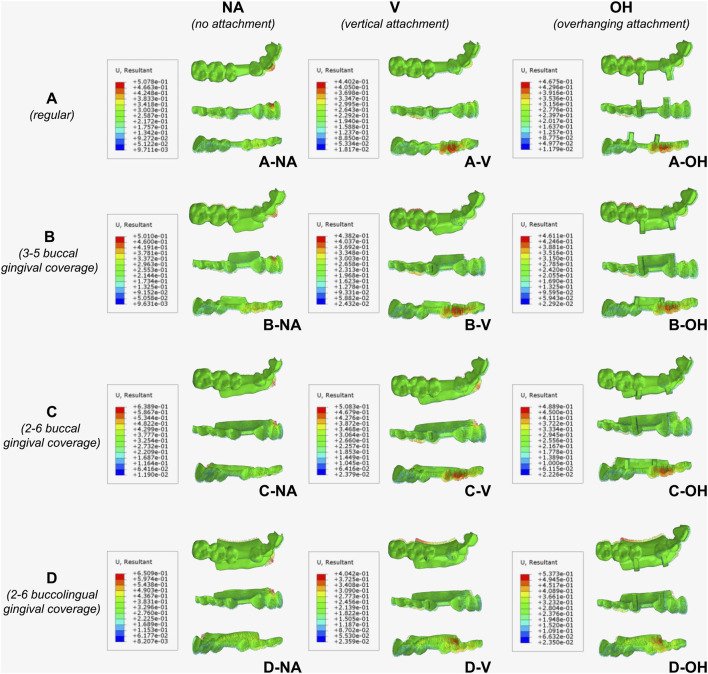
Heatmap of CA deformation in vector diagram, with occlusal, buccal, and lingual views. **(A)** Regular CA; **(B)** CA with buccal gingival coverage from 3 to 5; **(C)** CA with buccal gingival coverage from 2 to 6; **(D)** CA with buccolingual gingival coverage from 2 to 6.

The deformation of the CA was shown in Figure 8. The maximum deformation occurred at the labial side of lateral incisor in groups without attachments (NA), which was attributed to the compression caused by anchorage effect that endowed the mesial movement of canine, resulting in a labial protrusion. With attachments on 3 and 5, maximum deformation occurred around the attachment on canine, deformation of CA at the lateral incisor was reduced, and the deformation at the buccal side of molars became more pronounced. As evidenced by the upper limits of the heatmap scale, introduction of attachments reduced the overall maximum deformation of CA, indicating more evenly distributed orthodontic force. With buccal extended coverage on 2∼6, the overall maximum deformation of CA increased, while with buccolingual extended coverage on 2∼6, the overall maximum deformation of CA decreased except for the OH attachment group (D-OH). The buccolingual extension exerted a slight torsion along the dental arch curve towards the mesial direction. Moreover, upon increasing the lingual and gingival coverage, and the maximum deformation of the CA shifted to the lingual gingival extension under the first molar, resulting in a decrease in deformation of molar crowns and enhancing the retention of CA, thereby avoiding the occurrence of attachment debonding.

### 3.4 Stress distribution on CA

**FIGURE 9:**
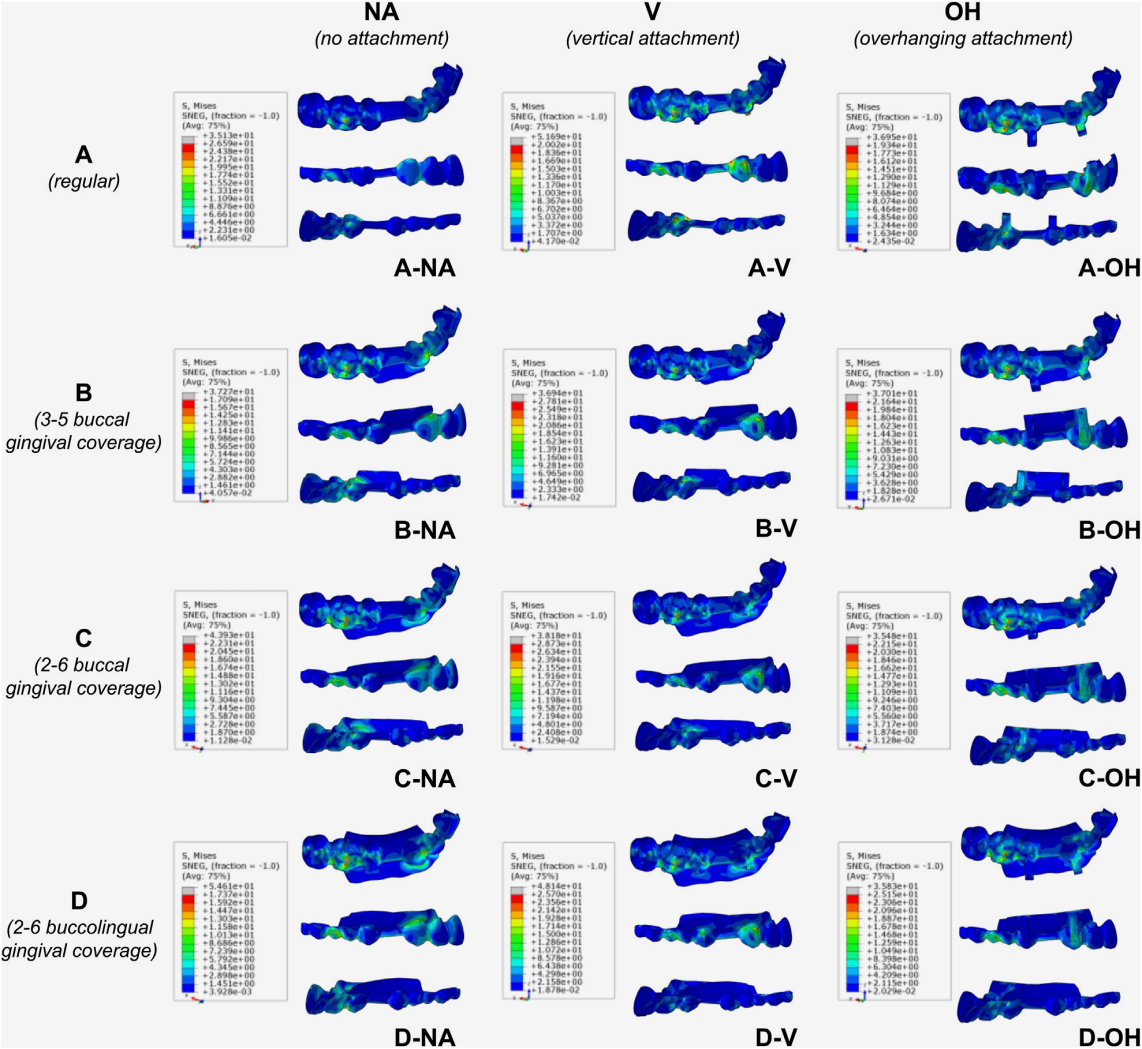
Von-Mises Stress distribution on CA. Red regions indicated the maximum stress. **(A)** Regular CA; **(B)** CA with buccal gingival coverage from 3 to 5; **(C)** CA with buccal gingival coverage from 2 to 6; **(D)** CA with buccolingual gingival coverage from 2 to 6.

Figure 9 indicated that an increase in maximum Von-Mises stress value was observed in CAs with no attachment (NA) as the gingival coverage enlarged (D > C > B > A). Comparing A-V with A-NA, the maximum Von-Mises stress values increased after the addition of a regular vertical attachment (V), while buccal extension of CA (B-V and C-V) resulted in decreased maximum Von-Mises stress to the level of A-N. Using regular vertical attachment (V), buccolingual extension (D-V) exhibited slightly increased stress compared to buccal extension (B-V and C-V). Notably, using OH attachment, there was no significant difference in the maximum Von-Mises stress values with or without extended gingival coverage, indicating the OH attachment could minimize stress concentration in CA. The membrane material employed in this simulation was PETG, with a yield strength of 45 MPa. All designs of CA met the material strength requirements, ensuring that no plastic deformation occurred during the wearing process.

## 4 Conclusion

This study first reported the biomechanical effects of overhanging attachment combined with partial gingival extension of CA trimline during closing extraction spaces. The results indicate that the overhanging attachment was more effective than the regular vertical attachment in achieving root-controlled canine movement. The highest orthodontic efficiency was observed when the overhanging attachment was combined with a buccolingual gingival extension of CA trimline on 2–6, while simultaneously avoiding excessive aligner deformation and stress concentration. This study provided theoretical bases for the clinical application of overhanging attachment. The partial gingival extension of the CA trimline proposed in this study demonstrated greater feasibility in clinical application compared to the full gingiva-covering trimline design. The methodology and findings presented here provided reference and guidance for the design of future orthodontic treatment.

## Data Availability

The original contributions presented in the study are included in the article/supplementary material, further inquiries can be directed to the corresponding authors.
